# Assessment approaches in undergraduate health professions education: towards the development of feasible assessment approaches for low-resource settings

**DOI:** 10.1186/s12909-024-05264-x

**Published:** 2024-03-20

**Authors:** Eva Mukurunge, Champion N. Nyoni, Lizemari Hugo

**Affiliations:** School of Nursing, Faculty of Health Sciences, University of the Free State, P.O. Box 339, Bloemfontein, 9300 South Africa

**Keywords:** Assessment, Health professions education, Mapping review, Undergraduate education

## Abstract

**Background:**

Feasible and effective assessment approaches to measuring competency in health sciences are vital in competency-based education. Educational programmes for health professions in low- and middle-income countries are increasingly adopting competency-based education as a strategy for training health professionals. Importantly, the organisation of assessments and assessment approaches must align with the available resources and still result in the fidelity of implementation. A review of existing assessment approaches, frameworks, models, and methods is essential for the development of feasible and effective assessment approaches in low-resource settings.

**Methods:**

Published literature was sourced from 13 electronic databases. The inclusion criteria were literature published in English between 2000 and 2022 about assessment approaches to measuring competency in health science professions. Specific data relating to the aims of each study, its location, population, research design, assessment approaches (including the outcome of implementing such approaches), frameworks, models, and methods were extracted from the included literature. The data were analysed through a multi-step process that integrated quantitative and qualitative approaches.

**Results:**

Many articles were from the United States and Australia and reported on the development of assessment models. Most of the articles included undergraduate medical or nursing students. A variety of models, theories, and frameworks were reported and included the Ideal model, Predictive Learning Assessment model, Amalgamated Student Assessment in Practice (ASAP) model, Leadership Outcome Assessment (LOA) model, Reporter-Interpreter-Manager-Educator (RIME) framework, the Quarter model, and the model which incorporates four assessment methods which are Triple Jump Test, Essay incorporating critical thinking questions, Multistation Integrated Practical Examination, and Multiple Choice Questions (TEMM) model. Additional models and frameworks that were used include the Entrustable Professional Activities framework, the System of Assessment framework, the Reporter-Interpreter-Manager-Educator (RIME) framework, the Clinical Reasoning framework (which is embedded in the Amalgamated Student Assessment in Practice (ASAP) model), Earl’s Model of Learning, an assessment framework based on the Bayer–Fetzer Kalamazoo Consensus Statement, Bloom's taxonomy, the Canadian Medical Education Directions for Specialists (CanMEDS) Framework, the Accreditation Council for Graduate Medical Education (ACGME) framework, the Dreyfus Developmental Framework, and Miller's Pyramid.

**Conclusion:**

An analysis of the assessment approaches, frameworks, models, and methods applied in health professions education lays the foundation for the development of feasible and effective assessment approaches in low-resource settings that integrate competency-based education.

**Trial registration:**

This study did not involve any clinical intervention. Therefore, trial registration was not required.

**Supplementary Information:**

The online version contains supplementary material available at 10.1186/s12909-024-05264-x.

## Background

Implementing competency-based education in health professions education within low-resource settings presents significant challenges. Competency-based education (CBE) is a model for designing and implementing education that focuses on the desired performance characteristics or outcomes of healthcare professionals [1]. The CBE model is set to improve student competence by predefining educational outcomes, also known as Entrustable Professional Activities, that support learning and teaching activities [2, 3]. The fundamental goal of CBE is to empower students with competencies in communication, collaboration, professionalism, and health advocacy, amongst others, to cultivate unique intellectual, emotional, and physical abilities so that they become successful in their professional lives, which will ultimately culminate in the improvement of patient care [1, 4]. However, the aspirations of CBE are rarely achieved in nursing practice in low-resource settings for a range of reasons, among which are poor understanding of what constitutes CBE, unclearly formulated competencies, poor implementation strategies, inadequately prepared educators, and unfeasible assessment methods [1, 5].

Implementors of the CBE model must ensure the feasible use of robust assessment methods that result in valid and reliable scores that can be used to provide feedback on learning and make decisions about student progression. In this paper, an assessment method refers to a technique employed to collect information on the performance of a student at a specific instance, for example, a multiple-choice examination [1, 6]. Conventional assessment approaches focus on end-of-course high-stakes examinations of knowledge and may be oblivious to authentic performance [7, 8]. These assessment approaches are mainly summative, may lack detailed feedback and may be limited in how they enable the monitoring of growth in student performance. These approaches are also not aligned with the aspirations of CBE in health professions education (HPE) [7, 9]. In this study, an assessment approach refers to a set of principles that guide the implementation of assessment in an educational programme The aspirations of CBE were spelt out by the competency-based medical education collaborators.

The competency-based medical education (CBME) collaborators established a CBME Core Components framework aimed at increasing fidelity in implementing CBME [5, 10]. There are five core components of the CBME framework: an outcomes competency framework; progressive sequencing of the outcomes; learning experiences that are tailored to the competencies in the CBME; teaching that is tailored to the competencies; and assessment following the programmatic assessment (PA) approach [5, 10]. Accordingly, PA should be embedded in the design and implementation of a CBE programme.

PA takes a longitudinal and holistic approach to assessment and emphasises the learning function of assessment by using an array of assessment methods to provide feedback and make plausible assessment decisions [11–13]. The PA approach is systematic since it encompasses planning with deliberate choices of assessment methods, scheduling, and feedback strategies [11, 14]. There are twelve principles that underpin the PA approach, which stand to reiterate that every individual assessment method has limitations and, if used alone to gather information on student performance, compromises will be made to reach pass or fail decisions [14]. In PA, information on student competence and progression is purposively and continually collected and analysed [3, 11, 15]. The concept of ‘data point’ applied in the PA approach refers to information on student performance that is collected from a single assessment method [8, 12]. Single data points are used in formative assessment to provide constructive feedback, which guides learning [13, 16]. The progressive accumulation of multiple data points becomes the premise of pass-or-fail decisions reached by a group of assessment experts [3]. According to the PA approach, the pass-or-fail decisions should not be reached by individuals, but rather by competence committees [14]. Programmatic assessment is instrumental in the implementation of a valid CBE programme since effective assessment is a strong force behind the authenticity of CBE [17].

The implementation of PA, however, is resource-intensive [12, 18]. Increased fidelity of PA implementation, therefore, requires a number of structures to be in place. There should be a well-established support structure for educators, a supportive administrative department in the institution, and an established group of experts who will make high-stakes assessment decisions affecting students’ progression in the programme. Additional aspects that should be in place include training workshops for educators, mentors, and preceptors in the clinical area, as well as timely and constructive feedback after each assessment and the use of multiple methods of assessment for the collection of data on student performance [11, 12, 18]. A large volume of data on student performance can be collected, given the multiple methods of assessment used in PA. Therefore, an information management system needs to be established [18]. Since no set number of data collection points is stipulated, institutions often quantitatively warrant saturation of information on student performance by setting a minimum requirement for the number of data points to be collected [19]. The minimum number of data points deemed adequate for saturation differs according to the institutional context [19].

The success of PA implementation is context-dependent [13]. Instances of successful PA implementation are sparse in low-resource contexts and skewed towards institutions in high-income countries [8, 14]. Canada, the United Kingdom, the United States of America, the Netherlands, New Zealand, and Australia are reported as having successfully implemented the PA approach in their undergraduate medical programmes [14]. One low-resourced country in Africa, Uganda, reports the successful implementation of PA [20]. There are various reasons that explain why the low uptake of PA approaches exists in low-resourced contexts, including resource disparities, poor leadership and institutional governance, and the limited adoption of CBE across settings [3]. The positive skew towards countries in Europe and North America may be due to the development, implementation, and review of PA led by assessment experts from HPE institutions in those regions [3, 21].

There is also an interplay of various factors that influence the fidelity of implementing PA. On the one hand, there are factors such as the political, economic, and social context of the institution, poor organisational culture, poor leadership engagement, poor support structures for educators, which appear to negatively influence implementation [13, 21]. On the other hand, factors such as robust leadership engagement, financial support, adequate support for educators, adequate human resources, and frequent workshops for educators are reported as quintessential to the successful implementation of this approach [18]. The outcome of this interplay of factors positions the implementation of PA as resource intensive and thus unachievable for institutions that aspire to implement CBE in resource-limited contexts [12]. Failure by HPE institutions in resource-limited settings to implement the recommended PA approach for authentic CBE programmes has various implications, which have ripple effects on the fidelity of CBE. The implications include, the adoption of feasible and conventional approaches to assessment, drifting away from the PA approach (which has a negative effect on the implementation of CBE), false positive results of CBE implementation, curriculum drift, and graduates who are not competent or ready for work in the health system [3].

There is thus a need to develop a defensible and feasible assessment approach that can be implemented in CBE programmes, especially in low-resource contexts. The developed assessment approach should aim to maintain a balance between the inherent characteristics of the CBE model and enabling factors in the educational context. This article focuses on a mapping review, which reports on the assessment approaches, frameworks, models, and methods related to the implementation of assessment in undergraduate HPE to inform the development of an assessment approach for institutions implementing CBE models in low-resource contexts.

## Methods

The review question for this study was:

*W* *hat* *is known about assessment approaches, frameworks, models, and methods in undergraduate health professions education?*

### Study design

The mapping review study design used for this research was structured to enable the collection of literature specific to the field of assessment approaches. The aim was to develop a better understanding of the different characteristics of the assessment approaches, frameworks, models, and methods used in undergraduate health professions education. The aim was also to identify potential gaps in previous research.

The mapping review followed a stepwise approach comprising five steps including searching and screening the literature, data extraction, and analysis and presentation of results.

#### Step 1: Searching the literature

Searching the literature involves developing a search strategy that comprises the search string and databases to be searched.

### Search string

The search string was determined by integrating keywords and synonyms gleaned from the review question.This was done through Boolean operators and modifiers. The search string was:

(assess* n2 (model* or framework* or theories or theory) and (educat* or train*) and (“health profession*” or “health science*” or nurs* or medical or clinical or medicine) and (undergraduate* or baccalaur*) and ti assess*.

### Databases

The search for literature was carried out in May 2022 and covered the period from January 2000 to June 2022. The start date of the early 2000s was chosen due to an increase in the adoption of the CBE approach at that time, which saw new assessment approaches [17]. Ten databases were accessed through the EBSCOhost interface by the first author in collaboration with an information specialist at the university library. Table 1 shows the databases accessed and publication records retrieved from each.

**Table 1:** Databases used and abstracts retrieved

Database	Publication records retrieved
MEDLINE	86
Academic Search Ultimate	42
CINAHL with Full Text	38
APA PsycInfo	22
Health Source: Nursing/Academic Edition	22
Africa-Wide Information	5
ERIC	10
CAB Abstracts	1
Communication & Mass Media Complete	1
Sociology Source Ultimate 1	1

#### Step 2: Screening the literature

The search yielded 228 records, which were reduced to 135 by the automatic deduplication process. A further manual deduplication yielded 121 publication records excluding 14 records. The following inclusion and exclusion criteria were then used to screen the remaining 121 publication records.

### Inclusion/exclusion criteria

#### Inclusion criteria

Studies included were peer-reviewed literature published on assessment approaches, strategies, theories, and methods in undergraduate programmes in health professions education.

#### Exclusion criteria

Studies were excluded if 1) their literature focused on assessment in organisations; 2) the content was about postgraduate education; 3) the assessment was in primary and secondary schools; or 4) the literature did not focus on HPEs and reviews.

## Results

The three authors then screened the 121 records against the inclusion/exclusion criteria based on their titles and abstracts. The authors screened these records independently and were blinded of their screening outcomes until a consensus meeting. Discussions among the authors on the screening outcomes were held, and any discrepancies were resolved. A total of ninety (*n* = 90) abstracts did not meet the inclusion criteria and were eliminated.

**Fig. 1:**
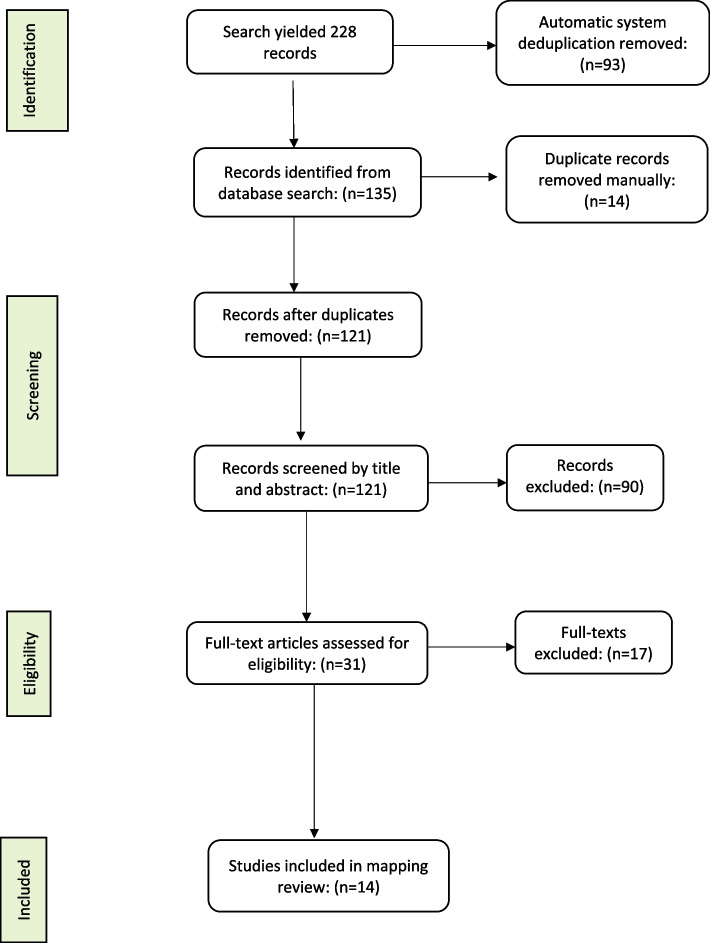
Prisma flow diagram of the mapping review

Full-text articles for the remaining thirty-one (*n* = 31) abstracts were retrieved, read, and screened individually by all three authors. A further seventeen (*n* = 17) articles that did not meet the inclusion criteria were discarded. The remaining fourteen (*n* = 14) full-text articles were included in this study, as shown in Fig. 1.

### Step 3: Data extraction

Data to answer the review question were extracted from the final fourteen full-text articles (*n* = 14). Data extraction was literatim. A Google form was designed for the data extraction. Data elements extracted from the fourteen articles included the year of publication, the country where conducted, aim of the study, population, study design, assessment models, frameworks, approaches, and methods. A summary of the extracted data can be viewed as a supplementary file in this article.

### Step 4: Data analysis

Data were analysed quantitatively. Frequencies were mainly used to analyse data about the year of publication and the country where the study was conducted. Descriptive data analysis was used on the aim of the study, population, design, assessment approaches, frameworks, models, and methods. The information gathered from the data analysis was used to inform the development of an assessment approach that can be utilised in institutions implementing CBE models in low-resource contexts.

### Step 5: Presentation of results

The aim of the study was, through a mapping review, to report on the assessment approaches, frameworks, models, and methods related to the implementation of assessment in undergraduate HPE to inform the development of an assessment approach for institutions implementing CBE models in low-resource contexts. The results of the mapping review are discussed next by providing information about the contextual backgrounds of the different articles retrieved in the review, the characteristics of the studies, and the factors that are essential for the development of a feasible assessment approach.

#### Contextual background

**Fig. 2:**
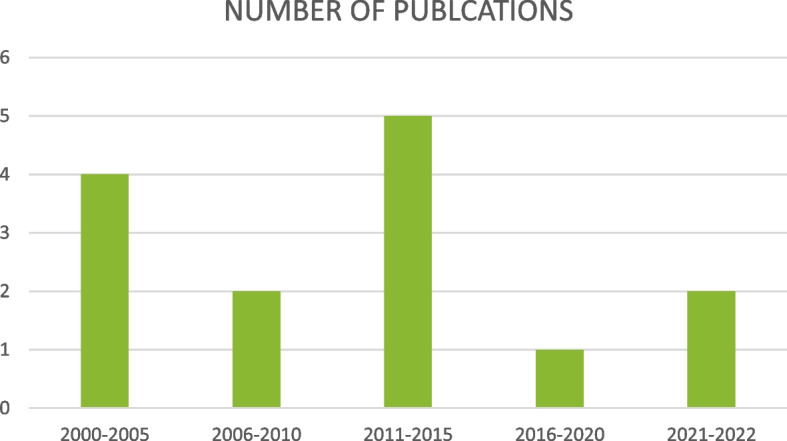
Number of publications per five-year period

The contextual background of the studies refers to the number of publications per 5-year period, the geographical distribution of the research output, and the target population of the publications. The years of publication ranged from the years 2000 to 2022, as illustrated in Fig. 2. Figure 2 also illustrates how research output was almost stagnant at 2 articles per each 5-year period over the 20-year period, with spikes to 4 and 5 over the period 2000–2005 and 2011–2015, respectively.

Countries where the studies were published and the number of publications over the 2000–2022 period are reflected in Table 2. The results show higher research output in high-income countries than in other countries.

**Table 2:** Geographical distribution of the articles

Country	Number of articles
Australia	3
Canada	1
India	3
Singapore	1
South Africa	1
United States of America	5

The target population of most of the articles (*n* = 11) were reported as undergraduate medical students, while the others (*n* = 3) focused on undergraduate nursing students. Regarding the study designs of the 14 articles, only Lafave, Katz, Vaughn, and Alberta (2013) reported a quasi-experimental design and the rest did not mention their study designs.

In terms of the aim of the studies, half of the articles (*n* = 7) reported on the implementation of assessment models that were utilised in their institutions [22–28]. The other articles (*n* = 7) reported on the development of, or proposal to develop assessment models to be implemented [29–35].

### Components essential for the development of a feasible assessment approach

The development of a feasible assessment approach requires background knowledge of the essential components of an assessment approach. Table 3 illustrates these components as assessment approaches, frameworks, models, and methods. The assessment models can be categorised into either purely clinical assessment models, or both theory and clinical assessment models.

**Table 3:**
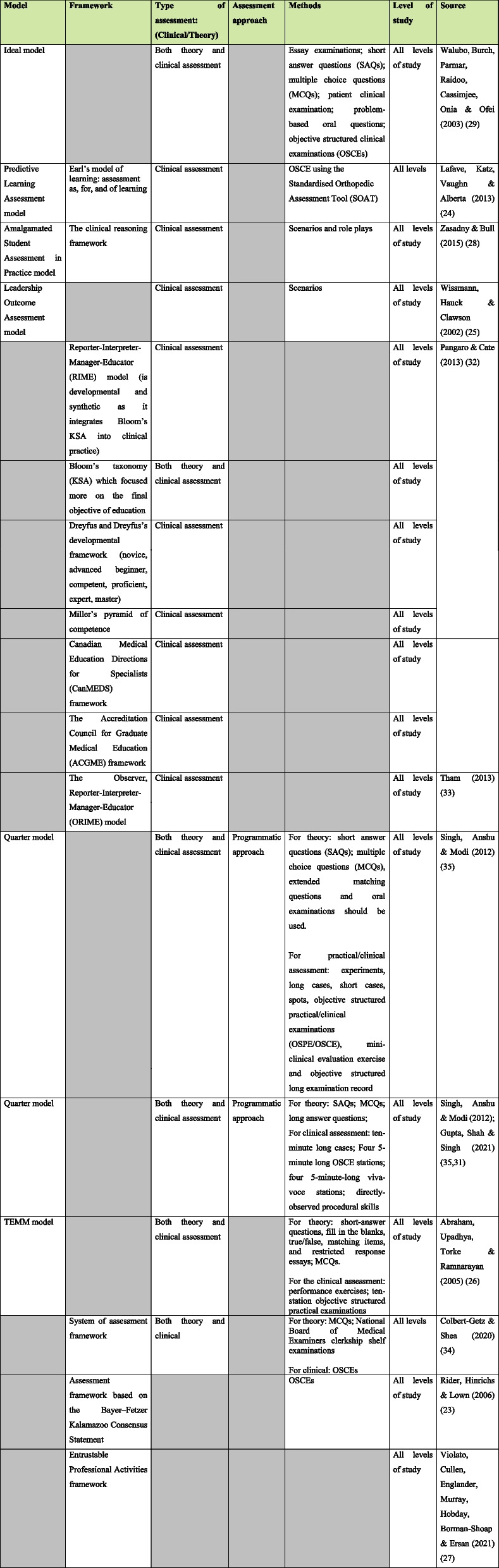
Assessment models, frameworks, type, approach and methods and study sample [23–29, 31–35]

NB: Grey scale sections were not reported

Table 3 shows that there are various assessment frameworks that are in use. Frameworks that guide assessment can be categorised into analytic, synthetic, and developmental frameworks. Examples of analytic frameworks are Bloom’s taxonomy, the CanMEDS Framework, and the ACGME framework. The analytic assessment frameworks take a specific approach in the assessment of learning outcomes where competencies are categorised into individual domains, for example, the psychomotor domain. Students are given feedback on their performance in each aspect of a specific task. The developmental framework is the Dreyfus Developmental Framework. The developmental assessment frameworks focus on the progress of students in developing skills through predetermined levels of competence. The synthetic frameworks are the RIME framework and Miller’s Pyramid. Synthetic frameworks allow for the holistic assessment of students as they apply all domains of competence in carrying out a task. One assessment approach, the programmatic assessment, was mentioned in the review.

A wide variety of assessment methods are listed in Table 3. These assessment methods include the OSCEs, scenarios, reflective reading logs, portfolios, laboratory reports, online discussion group submissions, essays, reports, projects, MCQs, problem-based oral examinations, structured short and essay questions, case studies and viva-voces. Assessment methods can be classified into theory methods and practical/clinical methods [35]. Theory assessment methods include SAQs, MCQs, extended matching questions, and oral examinations. Practical assessment methods include long cases, short cases, OSCEs, mini-clinical evaluation exercises, and objective structured long examination records.

## Discussion

The mapping review explored assessment approaches, frameworks, models, and methods in undergraduate HPE over the years 2000 to 2022 as a baseline for the development of an assessment approach. The developed assessment approach could be utilised by institutions in low-resource countries that wish to implement CBE models. Important to note here is that structured assessment processes are essential in CBE curricula. Generally, the mapping review revealed that there is limited discourse around the topic of assessment approaches in HPE. In the past 22 years, there has been minimal research output on this topic, and the research that has been published is generally skewed towards high-income countries.

Indeed, the geographic and economic orientation of the research used for the mapping review showed that the majority of publications were from high-income countries like the United States of America, New Zealand, Australia, Canada and Singapore. According to Govaerts et al. [3] there is marked success in the implementation of CBE in high-income countries (HICs), hence the higher publication output. Lema, Kraemer-Mbula and Rakas [36] also concluded that health education professionals in high-income countries can afford to implement various assessment models and publish their outcomes, unlike their counterparts in low-resource countries. Lema, Kraemer-Mbula and Rakas [36] reiterate that research on innovation is generally distributed along income lines and that even though research output on innovation in low- to middle-income countries (LMICs) has grown substantially in the past two decades, it is still skewed towards upper-middle-income countries like China.

Other reasons that explain why the majority of the research on assessment approaches in HPEs is from high-income countries include funding issues in low- and middle-income countries (LMICs), which hinder research output [37, 38]. Inadequate funds lead to poor information technology, unstable power supply, and the inaccessibility of libraries and journals [37]. There appears to be insufficient human capacity in research as well as few research mentors and role models, and a lack of a research culture, which can have a negative impact on research output in HPE institutions in low-income countries. Thus, although there is a significant amount of innovation taking place in HPE institutions in LMICs, there is still limited research output. There also seems to be insufficient networking among research communities in LMICs, meaning that support among researchers is lacking. Limited use of research evidence could, in turn, demotivate researchers to engage in further research. Unlike in HICs, students in LMICs are often introduced to research late into their academic journeys. In LMIC contexts, research is mostly introduced towards the end of a student’s undergraduate degree. Additionally, limited career options in research means that potential researchers may only implement research projects as partial fulfilment of their degrees and not as a career pathway trajectory. A further issue is the poor reception of research papers in reputable journals, which may also dampen the researcher spirit [38].

Research theory, however, does provide some insight into the ways in which research in LMICs can be better enabled. Factors that can enable research include: allowing for curricula innovation and high research output, keeping class sizes relatively small, the presence of specialised assessment experts who offer support to faculty, a collegial environment, a centralised funding system, outstanding information technology resources, state-of-the-art clinical simulation centres, a shared educational vision with the leadership of the institution, stakeholder involvement, input from other departments at the university that may have already successfully implemented CBE, a centralised governance structure, and educational consultants who support the programme [37].

This mapping review also revealed that there is some stagnation in the field of assessment in CBE. This stagnation could be linked to the curriculum innovation taking place within many HPE institutions, which means that they are yet to establish feasible assessment approaches in their contexts. To illustrate this point, half of the papers (*n* = 7) from the review reported on the development of, or proposals to develop assessment models, which are thus yet to be implemented [29–35]. HPE institutions still seem to be trying to find their footing in terms of assessment in CBE.

**Fig. 3:**
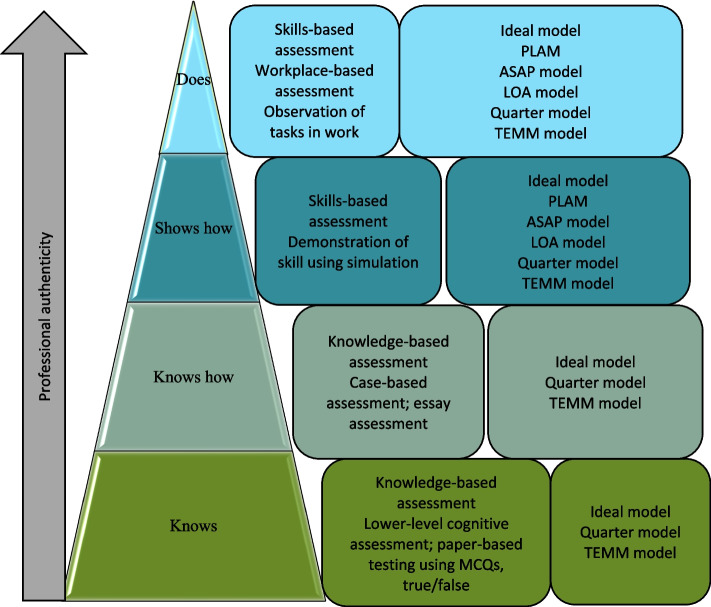
Adaptation of miller’s pyramid of clinical competence (1990)

The development of a feasible assessment approach should be supported by good assessment frameworks. The assessment approaches used for the assessment of competence in CBE can be structured around Miller’s competency pyramid as shown in Fig. 3 below [39, 40]. Miller’s Pyramid presents a framework that can be used to assess levels of clinical competence from cognitive levels of knowledge (knowing and knowing how), application of knowledge (showing), practical application of the knowledge in a practice setting (doing) [40]. Miller’s pyramid has become the beacon of assessment frameworks in CBE as it allows assessment of all facets of competence, which include knowledge, skills, and attitude. Miller’s pyramid divides the development of clinical competence into four hierarchical processes with knowledge at the lowest level, tested by written examinations and MCQs. The second level, application of the knowledge, is assessed by essays, clinical problem-solving exercises, and extended MCQs. The third level, clinical skills competency, is assessed by OSCEs. The final level, clinical performance, is assessed by direct observation in real clinical settings [40]. The use of frameworks in collaboration with assessment models will guide the development of an appropriate assessment.

Assessment models and frameworks in CBE are centred around students’ attainment of competency. Miller’s framework guides the assessment of competence in CBE [39] and how a student progresses from novice to expert in their academic growth, as illustrated in Fig. 3 above.

Assessment approaches are associated with the use of assessment methods in collecting data during the academic journey of the student. The choice of assessment method depends on the educational justification for using the method at a particular time [41]. Some authors believe that oral assessment methods, like presentations and viva voces, can be used to allow students to better express themselves and to give the assessor an opportunity to probe further in gaining a clearer picture of the student’s understanding of the content [42]. In terms of written assessments, however, a comparison between SAQs and MCQs may give a false impression of students’ performance. SAQs give students a chance to express their cognitive capabilities and errors [43]. However, the disadvantage of SAQs is that students focus on practising examination techniques rather than on having a full understanding of the principles of the subject matter. Hence, SAQs do not prepare students for the clinical tasks they will have to complete with patients, which require them to apply their medical knowledge [43]. Therefore, the necessity of a mix of assessment methods to ensure a comprehensive assessment of the student.

One assessment method that has gained popularity in medical and healthcare education since its introduction by Harden in 1975 is the OSCE [44]. The success of OSCE is based on the specific measurements that OSCE has, which are validity, reliability, feasibility, and credibility. The inherent strength of OSCE is its objectivity because examiner and patient variations are eliminated [45]. However, OSCEs require much planning and can be resource intensive in terms of budget for training examiners, remunerating simulated patients, and setting up multiple stations in contexts with large student numbers [45, 46].

The uptake and implementation of CBE in HPE institutions have not been flawless. This has negatively impacted the assessment strategies utilised [47]. The first flaw is often disagreement in what the terms ‘competence’ and ‘competency’ mean. This disagreement has led some nations to contextualise their understanding of the terms, which has, in turn, led to varied implementation strategies of CBE and its assessment strategies. Some countries have instituted CBE but later stepped back from some or all of their curriculum reform strategies. For example, Sweden partly unraveled its earlier CBE approach in 2011. England went on to replace its competency-based curriculum in 2014 [48], and Japan, Poland [49] and the Flemish community of Belgium [50] are said to have shifted back towards more discipline-focused curricula. All the evidence seems to paint a picture that CBE, and its associated assessment strategies, are not easy to implement. Therefore, HPE institutions that wish to adopt CBE might have to develop an assessment approach that is feasible in their context, yet fulfils the mandate of assessment in CBE.

There is also a discrepancy in the fidelity of implementation due to political influences as some countries rhetorically announce that they have joined the group of nations that have adopted CBE without adopting the same curriculum reform as those countries [47]. These countries have developed hybrids of the CBE curriculum. Most countries that have been unsuccessful in rolling out a CBE curriculum have altered the ideas of CBE to fit into their national political, economic, or cultural contexts. Deng and Peng [51] have shown how China’s competency framework is adapted to its Confucian and socialist context. The United States have combined their competency framework with pragmatism. The Swedish reform led to the meshing of content-based reforms with competency-based reforms. This hybridity in approaches to curricula supports the overarching argument of this study, which is to stress the need to develop an assessment approach that is feasible for implementation in low-resource settings.

## Conclusion

As HPE institutions adopt CBE, maintenance of the fidelity of the CBE is essential. To keep the fidelity of CBE implementation high, the PA approach has to be utilised in the assessment of and for learning. However, the reviewed literature has revealed that PA is a resource-intensive approach. As a result, institutions that cannot afford the implementation of PA will likely adopt CBE, but resort to affordable, traditional assessment methods. This mapping review set out to reveal the importance of establishing what factors are essential in developing an alternative and feasible assessment approach that fulfils the requirements of assessment in CBE to be used in low-resource settings. Based on the results of this mapping review, future research should seek to develop a more feasible assessment approach that can be used in CBE in those contexts where the implementation of PA is costly.

### Limitations

Time was one of the limiting factors in this research since it was part of a study qualification, which had to be completed within a specific time frame. Language limited the number of articles that could be accessed in the review since English-only articles were retrieved.

### Recommendations

Educators planning for the development of an assessment approach should consider a mapping review that includes other non-English articles to broaden the results. Assessment models, frameworks, and methods are essential in structuring the development of a new assessment approach. Therefore, educators should be guided by considering the selection of a model, framework, and assessment methods that are feasible in their context.

### Supplementary Information

**Supplementary Material 1.**

## Data Availability

The datasets generated and/or analysed during the current study have been included in the results section and are publicly available. A raw data file has been added in the supplementary material and is publicly accessible. The link to the raw data extraction tool is: https://docs.google.com/spreadsheets/d/1N410YOAj6KRKFauWm0-rsI5Hf0YMIXeK0wKWMKA_UyI/edit?usp=sharing

## Abbreviations

CBE: Competency-based education
HPE: Health profession education
MCQ: Multiple choice questions
RIME: Reporter-to-interpreter, to-manager/educator
TEMM: A model consisting of 4 assessment methods: the Triple Jump Test, essay incorporating critical thinking questions, Multi-station Integrated Practical Examination, and multiple-choice questions
OSCE: Objective structured clinical examinations
SAQ: Short answer questions
LMICs: Low- and middle-income countries
HICs: High-income countries
